# Should medical students be involved in the fight against the coronavirus (COVID-19) pandemic?

**DOI:** 10.11604/pamj.supp.2020.35.2.22773

**Published:** 2020-04-30

**Authors:** Dimitrios Moris, Efstathia Liatsou, Dimitrios Schizas

**Affiliations:** 1 First Department of Surgery, National and Kapodistrian University of Athens, Laikon General Hospital, Athens, Greece

**Keywords:** Coronavirus, COVID-19, medical students, pandemic

## To the editors of the Pan African Medical Journal

During public health crises such as the COVID-19 pandemic, healthcare systems are constantly challenged to increased demand of healthcare services and resources. As a result, the institutional capacity is stretched facing the obvious danger of becoming overloaded while the professional medical and nursing staff reserve is getting recruited or even redeployed to cover the increasing needs [1]. In most medical schools, students are actively involved in patient care, mainly coordinating medications and simple procedures, consulting with nurses and specialists, and updating patients and their families on the daily care plan. Moreover, they are familiar with basic documentation of care principles and skills. Of interest, it is well documented in the literature that senior medical students can achieve statistically higher exam scores in knowledge fields such as epidemiology and biologic behavior of infectious diseases such as the Middle East respiratory syndrome coronavirus (MERS-CoV) than students from other relative schools that makes them a useful asset to the medical teams [2]. On the contrary, it is shown that the involvement of medical students in the frontline of health crises such as natural disasters or infectious diseases can have a negative impact on their physical and mental health [3,4].

The dilemma between the obvious need of additional human resources and the risk of exposing unprepared, inexperienced and premature healthcare personnel to the challenges of COVID-19 pandemic is the main debate of our article. We believe that medical students, especially the senior ones consist of a reliable and trustworthy medical population that can be actively and independently utilized in the process of assisting the medical and nursing staff during the ongoing COVID-19 pandemic. Their recruitment should be done at a volunteering basis with main focus on assisting with the development and management of the so-called “yellow zone regulation” including the phone call center assistance, by providing people with necessary information about the various means of individual precaution or receive calls from patients with symptoms. The value of the triage of patients can be really beneficial for the healthcare system because medical students are trained well enough to differentiate the “mild” cases that will not mandate any further workup and can be managed with self-quarantine at home from those with severe symptoms who are in urgent need of medical assistance and possible hospitalization [5]. Also, students can be involved in the direct interaction and psychological support of patients and their families. Moreover, medical students can reliably help with basic administrative needs such as supervised documentation and ordering of medical exams, thus partially alleviating the workload towards of the medical house staff. Also, students could be involved in data collection, record updating and patient follow-up that can be invaluable in the era of urgent need for COVID related research. Finally, students can be a useful resource of assistance with social tasks such as childcare, pet care and household support services. In addition to these efforts, they can be involved in collecting PPEs, sewing masks and working with 3D printing.

We acknowledge that there is a different view advocating for no involvement of medical students and other inexperienced personnel in the fight against this epidemiological crisis. The main concerns are the ethical component of their involvement in patient care, since none of them are certified at their level of training as well as the psychological impact of such involvement. The truth is that the management of this pandemic is like a saga in uncharted waters. And we also know that the virus makes no discrimination between healthcare providers and general population, that is documented by the incidence and disease-related mortality among healthcare personnel [6]. On the contrary, we cannot overlook the increasing needs for human resources to deal with this crisis, especially since more and more healthcare workers should be periodically outside the hospital environment either because they are sick or to be protected from getting exposed. It is of paramount importance that any student involved in this fight against COVID-19 will have all resources needed to maintain well-being such as clear task expectations and logistics, readily available supervision and measures to maintain their safety and hygiene, including practicing social distancing and not engaging in childcare unless they have been away from possible exposures in the clinical setting for at least 14 days.

## Conclusion

We acknowledge the unprecedented times that the society and workers in healthcare are experiencing. During these times, we believe that medical students can be a useful pool of efficient and trustworthy human resource to be wisely utilized against this pandemic. The quality of their training as well as their moral and professionalism are invaluable skills at a time of crisis. Nonetheless, their safety should remain a matter of high priority.

## Competing interests

The authors declare no competing interests.
